# Advanced Adenoma and Long-Term Risk of Colorectal Cancer, Cancer-Related Mortality, and Mortality

**DOI:** 10.1001/jamanetworkopen.2024.59703

**Published:** 2025-02-13

**Authors:** Aasma Shaukat, Paolo Goffredo, Jack M. Wolf, Kyle Rudser, Timothy R. Church

**Affiliations:** 1 Division of Gastroenterology and Hepatology, Department of Medicine, New York University Grossman School of Medicine, New York, New York; 2 Division of Colon and Rectal Surgery, Department of Surgery, University of Minnesota, Minneapolis; 3 Division of Biostatistics and Health Data Science, University of Minnesota School of Public Health, Minneapolis; 4 Division of Environmental Health Sciences, University of Minnesota School of Public Health, Minneapolis

## Abstract

This cohort study investigates the association of adenomas and advanced adenomas with colorectal cancer (CRC) incidence and mortality and all-cause mortality.

## Introduction

Colonoscopy is effective for reducing the risk of colorectal cancer (CRC) through detection of CRC and removal of adenomatous polyps. Studies have reported the association of advanced adenomas with increased risk of CRC and CRC-related death over the following 5 to 15 years compared with having no advanced adenomas.^1,2^ The association of nonadvanced adenomas with CRC incidence and mortality after colonoscopy is less clear.^1,2,3^ However, to our knowledge, no studies have evaluated the association of adenomas and advanced adenomas with all-cause mortality. We aimed to investigate the association of nonadvanced and advanced adenomas with CRC incidence and mortality and all-cause mortality compared with a negative colonoscopy among participants of the Minnesota Colon Cancer Control Study.

## Methods

The University of Minnesota Institutional Review Board approved the Minnesota Colon Cancer Control Study and this cohort study and waived informed consent because this study uses existing data collected with informed consent. STROBE reporting guidelines were followed. Data are from a multicenter, prospective cohort study of participants in the Minnesota fecal occult blood randomized clinical trial,^4^ which included 46 551 males and females aged 45 years and older, 10 584 of whom underwent colonoscopy after a positive fecal occult blood test. Colonoscopy findings were categorized as advanced adenoma (≥1 cm, high-grade dysplasia, or tubule-villous or villous histology), nonadvanced adenoma (<1 cm without advanced histology), or no adenoma. Cumulative incidence of CRC (competing risk of mortality), CRC mortality (competing risk of other-cause mortality), and overall mortality were estimated using Nelson-Aalen and Kaplan-Meier methods. Data were collected between 1975 and December 2013. Analysis was conducted between November 2023 and November 2024. Differences in incidence and mortality were compared using Fine-Gray and Cox estimators while adjusting for age at colonoscopy and sex. All statistical tests were 2-tailed at a significance level of .05. Statistical analyses were performed using R statistical software version 4.4.1 (R Project for Statistical Computing).

## Results

Among 10 584 participants who underwent colonoscopy (5298 male [50.1%]; mean [SD] age, 69 [8] years), the 20-year cumulative incidence of CRC for those with normal examinations, nonadvanced adenomas, and advanced adenomas was 1.8% (95% CI, 1.5%-2.1%), 3.9% (95% CI, 2.7%-5.4%), and 5.5% (95% CI, 4.1%-7.2%), respectively. Participants with nonadvanced (subdistribution hazard ratio [SHR], 2.24; 95% CI, 1.54-3.24; *P* < .001) and advanced (SHR, 3.24; 95% CI, 2.32-4.52; *P* < .001) adenomas were significantly more likely to develop CRC compared with those with no adenomas. Compared with participants with no adenomas, those with advanced adenomas were at significantly increased risk of CRC mortality (SHR, 2.20; 95% CI, 1.36-3.57; *P* < .001) and all-cause mortality (HR, 1.12; 95% CI, 1.03-1.21; *P* = .005), while participants with nonadvanced adenomas did not have a statistically significant increase in CRC mortality or all-cause mortality (Figure, A-C; Table).

**Figure.**
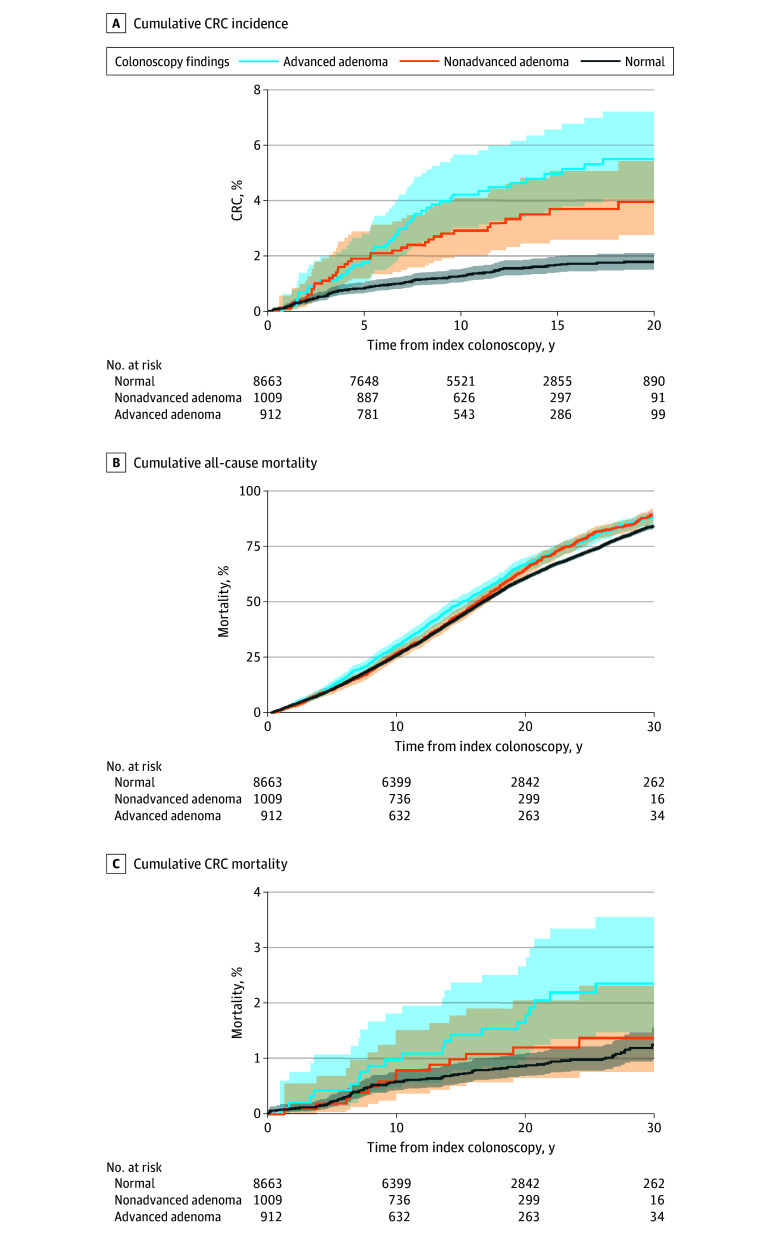
Cumulative Colorectal Cancer (CRC) Mortality, All-Cause Mortality, and CRC Incidence

**Table.** Association of Colonoscopy and Patient Characteristics With Outcomes

Characteristic	CRC incidence^a^		CRC mortality^a^		All-cause mortality^a^	
	SHR (95% CI)	*P* value	SHR (95% CI)	*P* value	HR (95% CI)	*P* value
Colonoscopy findings severity						
No findings	1 [Reference]	NA	1 [Reference]	NA	1 [Reference]	NA
Nonadvanced adenoma	2.24 (1.54-3.24)	<.001	1.28 (0.72-2.28)	.40	1.07 (1.00-1.16)	.06
Advanced adenoma	3.24 (2.32-4.52)	<.001	2.20 (1.36-3.57)	.001	1.12 (1.03-1.21)	.005
Age at colonoscopy, per 5-y increase	1.08 (1.00-1.16)	.07	1.11 (1.00-1.23)	.06	1.71 (1.68-1.74)	<.001
Male vs female	0.93 (0.71-1.21)	.58	0.71 (0.50-1.02)	.06	1.56 (1.49-1.63)	<.001

Abbreviations: CRC, colorectal cancer; SHR, subdistribution hazard ratio; HR, hazard ratio; NA, not applicable.

^a^ Adjusted for age at colonoscopy and sex.

## Discussion

In this cohort study, we found that over long-term follow-up, participants with nonadvanced and advanced adenomas at colonoscopy were at significantly increased risk of developing CRC compared with those with no adenomas. Participants with advanced adenomas were at increased risk for CRC mortality and all-cause mortality.

Our findings of increased risk of CRC incidence and mortality among individuals with advanced adenomas is similar to other findings.^1,2^ To our knowledge, our finding of increased risk of all-cause mortality among those with advanced adenomas has not been previously reported. Given that CRC mortality is a fraction of all-cause mortality, it is unlikely to explain our findings. Possible explanations include shared risk factors, such as smoking, high body mass index or other health behaviors, or genetic predispositions associated with development of advanced adenomas and other causes of mortality. In particular, we found male sex to be a risk factor associated with mortality; this suggests a role for high-risk lifestyle behaviors, such as occupational behaviors or those that are a consequence of occupation that may differ between males and females. Study limitations include an older time period for colonoscopies and lack of surveillance colonoscopy information. Our study underscores the importance of surveillance colonoscopy for individuals with advanced and nonadvanced adenomas and supports current recommendations of more frequent surveillance for those with advanced adenomas.

## Notes

Supplement.
Data Sharing Statement

## References

[1] Click B, Pinsky PF, Hickey T, Doroudi M, Schoen RE. Association of colonoscopy adenoma findings with long-term colorectal cancer incidence. JAMA. 2018;319(19):2021-2031. doi:10.1001/jama.2018.5809 29800214 PMC6583246

[2] Løberg M, Kalager M, Holme Ø, Hoff G, Adami HO, Bretthauer M. Long-term colorectal-cancer mortality after adenoma removal. N Engl J Med. 2014;371(9):799-807. doi:10.1056/NEJMoa1315870 25162886

[3] Dubé C, Yakubu M, McCurdy BR, . Risk of advanced adenoma, colorectal cancer, and colorectal cancer mortality in people with low-risk adenomas at baseline colonoscopy: a systematic review and meta-analysis. Am J Gastroenterol. 2017;112(12):1790-1801. doi:10.1038/ajg.2017.360 29087393

[4] Shaukat A, Mongin SJ, Geisser MS, . Long-term mortality after screening for colorectal cancer. N Engl J Med. 2013;369(12):1106-1114. doi:10.1056/NEJMoa1300720 24047060

