# Supplementary material for: Advanced Adenoma and Long-Term Risk of Colorectal Cancer, Cancer-Related Mortality, and Mortality
Source: JAMA Netw Open. 2025 Feb 13;8(2):e2459703. doi: 10.1001/jamanetworkopen.2024.59703 (PMC11826353; doi:10.1001/jamanetworkopen.2024.59703)
Supplement: Supplement. — Data Sharing Statement [file jamanetwopen-e2459703-s001.pdf]

Data Sharing Statement

Shaukat. Advanced Adenoma and Long-Term Risk of Colorectal Cancer, Cancer-Related Mortality, and Mortality. *JAMA Netw Open*. Published online February 13, 2025. doi:10.1001/jamanetworkopen.2024.59703

Data
Data available: No
